# Genetic alterations on chromosome 16 and 17 are important features of ductal carcinoma in situ of the breast and are associated with histologic type

**DOI:** 10.1038/sj.bjc.6693372

**Published:** 1999-12

**Authors:** C B J Vos, N T ter Haar, C Rosenberg, J L Peterse, A-M Cleton-Jansen, C J Cornelisse, M J van de Vijver

**Affiliations:** Department of Pathology andDepartment of Cytometry and Cytochemistry, Leiden University Medical Center, PO Box 9600, Leiden, RC 2300, The Netherlands; Department of Pathology, Netherlands Cancer Institute, Plesmanlaan 121, Amsterdam, CX 1066, The Netherlands

**Keywords:** DCIS, LOH, amplification, CGH, Southern blot analysis

## Abstract

We analysed the involvement of known and putative tumour suppressor- and oncogene loci in ductal carcinoma in situ (DCIS) by microsatellite analysis (LOH), Southern blotting and comparative genomic hybridization (CGH). A total of 78 pure DCIS cases, classified histologically as well, intermediately and poorly differentiated, were examined for LOH with 76 markers dispersed along all chromosome arms. LOH on chromosome 17 was more frequent in poorly differentiated DCIS (70%) compared to well-differentiated DCIS (17%), whereas loss on chromosome 16 was associated with well- and intermediately differentiated DCIS (66%). For a subset we have done Southern blot- and CGH analysis. C-*erb*B2/*neu* was amplified in 30% of poorly differentiated DCIS. No amplification was found of c-*myc*, mdm2, *bek*, *flg* and the epidermal growth factor (EGF)-receptor. By CGH, most frequent alterations in poorly differentiated DCIS were gains on 8q and 17q22–24 and deletion on 17p, whereas in well-differentiated DCIS amplification on chromosome 1q and deletion on 16q were found. In conclusion, our data indicates that inactivation of a yet unknown tumour suppressor gene on chromosome 16q is implicated in the development of most well and intermediately differentiated DCIS whereas amplification and inactivation of various genes on chromosome 17 are implicated in the development of poorly differentiated DCIS. Furthermore these data show that there is a genetic basis for the classification of DCIS in a well and poorly differentiated type and support the evidence of different genetic routes to develop a specific type of carcinoma in situ of the breast. © 1999 Cancer Research Campaign


*British Journal of Cancer* (1999) **81**(8), 1410–1418
© 1999 Cancer Research Campaign
Article no. bjoc.1999.0860

### Genetic alterations on chromosome 16 and 17 are important features of ductal carcinoma in situ of the breast and are associated with histologic type

**CBJ Vos[1], NT ter Haar[1], C Rosenberg[2], JL Peterse[3], A-M Cleton-Jansen[1], CJ Cornelisse[1] and MJ van de Vijver[1,3]**

[1]Department of Pathology and [2]Department of Cytometry and Cytochemistry, Leiden University Medical Center, PO Box 9600, 2300 RC, Leiden, The Netherlands; [3]Department of Pathology, Netherlands Cancer Institute, Plesmanlaan 121, 1066 CX, Amsterdam, The Netherlands

**Summary** We analysed the involvement of known and putative tumour suppressor- and oncogene loci in ductal carcinoma in situ (DCIS) by microsatellite analysis (LOH), Southern blotting and comparative genomic hybridization (CGH). A total of 78 pure DCIS cases, classified histologically as well, intermediately and poorly differentiated, were examined for LOH with 76 markers dispersed along all chromosome arms. LOH on chromosome 17 was more frequent in poorly differentiated DCIS (70%) compared to well-differentiated DCIS (17%), whereas loss on chromosome 16 was associated with well- and intermediately differentiated DCIS (66%). For a subset we have done Southern blot- and CGH analysis. C-*erb*B2/*neu* was amplified in 30% of poorly differentiated DCIS. No amplification was found of c-*myc*, mdm2, *bek*, *flg* and the epidermal growth factor (EGF)-receptor. By CGH, most frequent alterations in poorly differentiated DCIS were gains on 8q and 17q22–24 and deletion on 17p, whereas in well-differentiated DCIS amplification on chromosome 1q and deletion on 16q were found. In conclusion, our data indicates that inactivation of a yet unknown tumour suppressor gene on chromosome 16q is implicated in the development of most well and intermediately differentiated DCIS whereas amplification and inactivation of various genes on chromosome 17 are implicated in the development of poorly differentiated DCIS. Furthermore these data show that there is a genetic basis for the classification of DCIS in a well and poorly differentiated type and support the evidence of different genetic routes to develop a specific type of carcinoma in situ of the breast.

© 1999 Cancer Research Campaign

**Keywords:** DCIS; LOH; amplification; CGH; Southern blot analysis

A variety of genetic alterations have been identified in invasive breast cancer. The most frequent aberrations include amplifications of oncogenes, mutations in p53 and loss of heterozygosity (LOH) at a number of other but still putative tumour suppressor genes. The multitude of genetic changes so far has precluded the construction of a molecular progression model comparable to that for colorectal development (Vogelstein et al, 1988). It seems likely, therefore, that breast cancer not only clinico-pathologically, but also genetically is a heterogeneous disease. The clinico-pathological heterogeneity is already manifest in its histologically recognizable precursor lesion, ductal carcinoma in situ (DCIS). DCIS represents a proliferation of malignant cells within the ducts and lobules of the breast. Recently, several histological classifications have been proposed (Tavassolli et al, 1992; Holland et al, 1994; Silverstein et al, 1995; Scott et al, 1997). In all of these classifications DCIS is divided primarily on the basis of nuclear grade and/or necrosis, while architecture is given secondary consideration. The classifications differ to some degree, but all subdivide DCIS into three main subtypes: well-, intermediately and poorly differentiated. The relevance of implementation of biological markers to facilitate the histological classification of DCIS is supported by several studies (Bobrow et al, 1994; Zafrani et al, 1994; Mack et al, 1997). These studies have shown that poorly differentiated DCIS predominantly lack the oestrogen (ER) and progesterone receptor (PR), have a high proliferative rate, exhibit aneuploidy and c-*erb*B2/*neu* and p53 overexpression. In contrast, well-differentiated DCIS are often ER- and PR-positive, have a low proliferative rate and rarely show c-*erb*B2/*neu* and p53 overexpression.

In DCIS, three allelotyping studies have previously identified chromosomal loci with common allelic loss in DCIS. In all studies it was found that LOH on 16q, 17p and 17q are the most frequently found alterations in DCIS (Aldaz et al, 1995; Radford et al, 1995; Fujii et al, 1996).

Gene amplifications in DCIS have been studied by Southern blot analysis, fluorescent in situ hybridization (FISH), bright field in situ hybridization (BRISH) or comparative genomic hybridization (CGH). FISH revealed amplification of c-*erb*B2/*neu* in 30% of the DCIS cases (Murphy et al, 1995; Coene et al, 1997). By Southern blot analysis and BRISH, cyclin D1 gene amplification was found present in 12% of DCIS (Vos et al, submitted) and by FISH in 18% of DCIS (Simpson et al, 1997). Two CGH studies were done recently for nine and five cases of DCIS respectively (James et al, 1997; Kuukasjarvi et al, 1997). The most frequently found alterations were gains of 1q, 6q, 8q, 17q, 19q, 20p, 20q and Xq as well as losses of 13q, 14q, 16q, 17p and 22q.

In the present comprehensive study we aimed to obtain an insight of the genetic changes occurring in the histologically different types of DCIS and to explore whether specific genetic alterations are associated with specific histologic types of DCIS.

*Received 25 November 1998*
*Revised 26 April 1999*
*Accepted 13 May 1999*

*Correspondence to:* MJ van de Vijver

**Table 1** Polymorphic markers and the percentage of LOH observed in DCIS allelotyping (het=heterozygosity frequency based on CEPH data)

| Chromosome | Marker | Het. | LOH | % |
|---|---|---|---|---|
| 1p21 | AMY2B | 0.68 | 0/35 | |
| 1q21–23 | APOA2 | 0.74 | 0/35 | |
| 1q21–23 | D1S104 | 0.76 | 2/47 | 4.2 |
| 1q32–q44 | D1S103 | 0.88 | 1/51 | 1.9 |
| 2p25–p24 | TPO | 0.67 | 0/32 | |
| 2q33 | CTLA4 | 0.92 | 0/48 | |
| 3p24.4–p22 | D3S11 | 0.93 | 5/61 | 8.2 |
| 3p24.2–p22 | D3S1768 | 0.73 | 3/45 | 6.7 |
| 3p22–p14 | D3S2456 | 0.81 | 7/55 | 12.7 |
| 3p24 | D3S1300 | 0.83 | 4/31 | 12.9 |
| 3pter–p24 | D3S1244 | 0.62 | 2/28 | 7.1 |
| 3q26–q26.3 | GLUT2 | 0.91 | 5/45 | 11.1 |
| 3q27–q28 | D3S196 | 0.67 | 6/58 | 10.3 |
| 4p21.1–p14 | D4S174 | 0.92 | 0/53 | |
| 5p | D5S392 | 0.92 | 0/57 | |
| 5q11.1–q13.3 | D5S107 | 0.82 | 0/49 | |
| 6p22 | D6S105 | 0.87 | 0/58 | |
| 6p24–p22 | D6S1279 | 0.86 | 0/52 | |
| 6p24–P22 | D6S1955 | 0.75 | 0/51 | |
| 6q | D6S1010 | 0.75 | 3/38 | 7.9 |
| 6q13–q21.1 | D6S251 | 0.78 | 2/52 | 3.8 |
| 6q26–q27 | IGFR2 | 0.42 | 3/25 | 12.0 |
| 7p15–p21 | D7S488 | 0.85 | 0/52 | |
| 7q31-qter | D7S550 | 0.83 | 0/49 | |
| 8p22–p12 | D8S1130 | 0.93 | 14/64 | 21.8 |
| 8p21–p12 | GATA119CO6 | | 8/51 | 15.6 |
| 9p21 | D9S43 | 0.83 | 0/51 | |
| 9q | D9S53 | 0.87 | 0/57 | |
| 10pter–p11.2 | D10S89 | 0.80 | 0/48 | |
| 10q11.2-qter | D10S109 | 0.71 | 0/39 | |
| 11pter–p11.2 | D11S875 | 0.90 | 2/59 | 3.4 |
| 11p15 | D11S1999 | 0.76 | 0/52 | |
| 11q | D11S897 | 0.84 | 8/54 | 14.8 |
| 11q22 | D11S35 | 0.88 | 8/57 | 14.0 |
| 11q23.3-qter | D11S836 | 0.70 | 3/42 | 7.1 |
| 12pter–p11.2 | D12S59 | 0.81 | 0/45 | |
| 12q23-qter | PLA2 | 0.73 | 0/38 | |
| 12q22-qter | D12S79 | 0.96 | 0/60 | |
| 13q12.1 | D13S289 | 0.74 | 2/27 | 7.4 |
| 13q14.1–14.3 | D13S153 | 0.82 | 4/34 | 11.8 |
| 13q12.3 | D13S260 | 0.78 | 2/38 | 5.3 |
| 13q11 | D13S175 | 0.77 | 4/52 | 7.7 |
| 13q12.1 | D13S290 | 0.46 | 2/29 | 6.9 |
| 13q | D13S321 | 0.78 | 3/38 | 7.9 |
| 14q11.2 | D14S50 | 0.77 | 0/41 | |
| 14q32.1-qter | D14S51 | 0.77 | 0/43 | |
| 15pter-qter | D15S1232 | 0.83 | 0/25 | |
| 15q25-qter | D15S87 | 0.87 | 0/47 | |
| 16p13.3 | HBAP1 | 0.76 | 6/45 | 13.3 |
| 16q21 | D16S265 | 0.73 | 9/33 | 27.2 |
| 16q21 | D16S503 | 0.81 | 8/28 | 28.5 |
| 16q22.1 | D16S512 | 0.76 | 16/48 | 33.3 |
| 16q22.1-qter | D16S2624 | 0.85 | 21/50 | 42.0 |
| 16q22.1-qter | D16S752 | 0.92 | 25/59 | 42.3 |
| 16q23.2 | D16S266 | 0.60 | 9/31 | 29.0 |
| 16q24.2–q24.3 | D16S1320 | | 20/53 | 37.7 |
| 16q24.3 | D16S305 | 0.82 | 9/33 | 27.2 |
| 17pter-qter | D17S969 | 0.72 | 16/51 | 31.3 |
| 17pter-qter | D17S1537 | 0.78 | 15/37 | 40.5 |
| 17p13.3 | D17S513 | 0.89 | 10/29 | 34.4 |
| 17p13.1 | TP53 | | 19/61 | 31.1 |
| 17p12 | D17S520 | 0.77 | 5/18 | 27.7 |
| 17q12–q22 | D17S579 | 0.87 | 14/50 | 28.0 |
| 17q21 | D17S855 | 0.82 | 15/48 | 31.2 |
| 17q23 | D17S588 | 0.85 | 16/45 | 35.5 |
| 18 | D18S46 | 0.80 | 3/52 | 5.8 |
| 18q11 | D18S34 | 0.81 | 3/46 | 6.5 |
| 19p13.3 | D19S177 | 0.82 | 0/36 | |
| 19q12–q13.2 | APOC2 | 0.80 | 0/42 | |
| 20p12 | D20S66 | 0.78 | 0/41 | |
| 20q13.3 | CSTP1 | 0.61 | 0/24 | |
| 21q22.3 | D21S156 | 0.92 | 0/48 | |
| 22q13 | CYP2D | 0.80 | 4/57 | 7.0 |
| Xp22.3 | Kallman | 0.61 | 0/39 | |
| Xq21.1–q23 | DXS454 | 0.75 | 1/52 | 1.9 |

### MATERIALS AND METHODS

### Loss of heterozygosity

#### *Selection of tumour material and DNA isolation*

For the LOH analysis we obtained 78 cases of pure DCIS from the archives of the Departments of Pathology of the Netherlands Cancer Institute (NCI), Leiden University Medical Center (LUMC) and University Hospital Nijmegen. The cases were selected on the basis of histological type and classified as well-differentiated ($n$ = 26), intermediately differentiated ($n$ = 17) and poorly differentiated ($n$ = 35) according to the classification proposed by Holland et al (1994). Briefly, the histologic classification of DCIS can be summarized as follows: poorly differentiated DCIS is composed of cells with pleomorphic nuclei varying in size and shape with irregular contours, coarse chromatin and prominent nuclei. Frequent mitosis and extensive 'comedo' necrosis centrally in ducts are usually present. The cells have no tendency towards polarization resulting in a solid growth pattern. Intermediately differentiated DCIS is characterized by cells with moderately pleomorphic, slightly larger nuclei than well differentiated DCIS. The cells show a definite tendency towards polarization, i.e. an orientation of the apical side towards a lumen, resulting in trabecular or cribriform pattern. Well-differentiated DCIS is characterized by cells with monomorphic, equally sized nuclei with smooth nuclear membrane, uniform, fine chromatin and inconspicuous nuclei. Architectural differentiation is evidenced by pronounced polarization resulting in cribriform, micropapillary and clinging growth patterns. The mitotic rate is low and necrosis is hardly ever seen. In Figure 1, typical examples of well-, intermediately and poorly differentiated DCIS are shown. All cases were histologically classified by two pathologists (JL and MvV) independently. In case of disagreement, agreement was reached by looking at the slide together. To exclude invasion, the lesion was fully embedded or at least eight sections were taken.

For each case tissue blocks containing at least 20 ducts with DCIS were selected and four sections of 25 μm were deparaffinized, stained with haematoxylin and eosin (H&E) and micro-dissected under an inverted microscope using a 22 gauge needle. All in situ foci were selectively dissected, pooled and used for DNA isolation. Paraffin-embedded, tumour-negative lymph nodes or normal breast tissue isolated from a different tissue-block, were used to provide constitutional DNA from each patient. Tissue was digested in 500 μl DNA extraction buffer (100 mM sodium chloride, 10 mM Tris–HCl (pH = 8.0), 25 mM EDTA (pH = 8.0) and 0.5% sodium dodecyl sulphate (SDS)) and incubated at

© 1999 Cancer Research Campaign

**Table 2** Association of histologic type of DCIS with loss of heterozygosity on chromosome 16 and 17 in respectively 70 and 72 cases of DCIS (*P*-values given were calculated using the $\chi^2$ test)

| | LOH 16q | Retention 16q | % LOH | LOH whole chrom. 17 | LOH 17p | LOH 17q | Retention 17 | % LOH |
|---|---|---|---|---|---|---|---|---|
| Well-differentiated DCIS | 16 | 7 | 70 | 0 | 1 | 3 | 19 | 17 |
| Intermediately differentiated DCIS | 10 | 6 | 63 | 1 | 3 | 6 | 6 | 63 |
| Poorly differentiated DCIS | 12 | 19 | 39 | 6 | 11 | 6 | 10 | 70 |
| | P-value: 0.037 | | | P-value: 0.0002 | | | | |

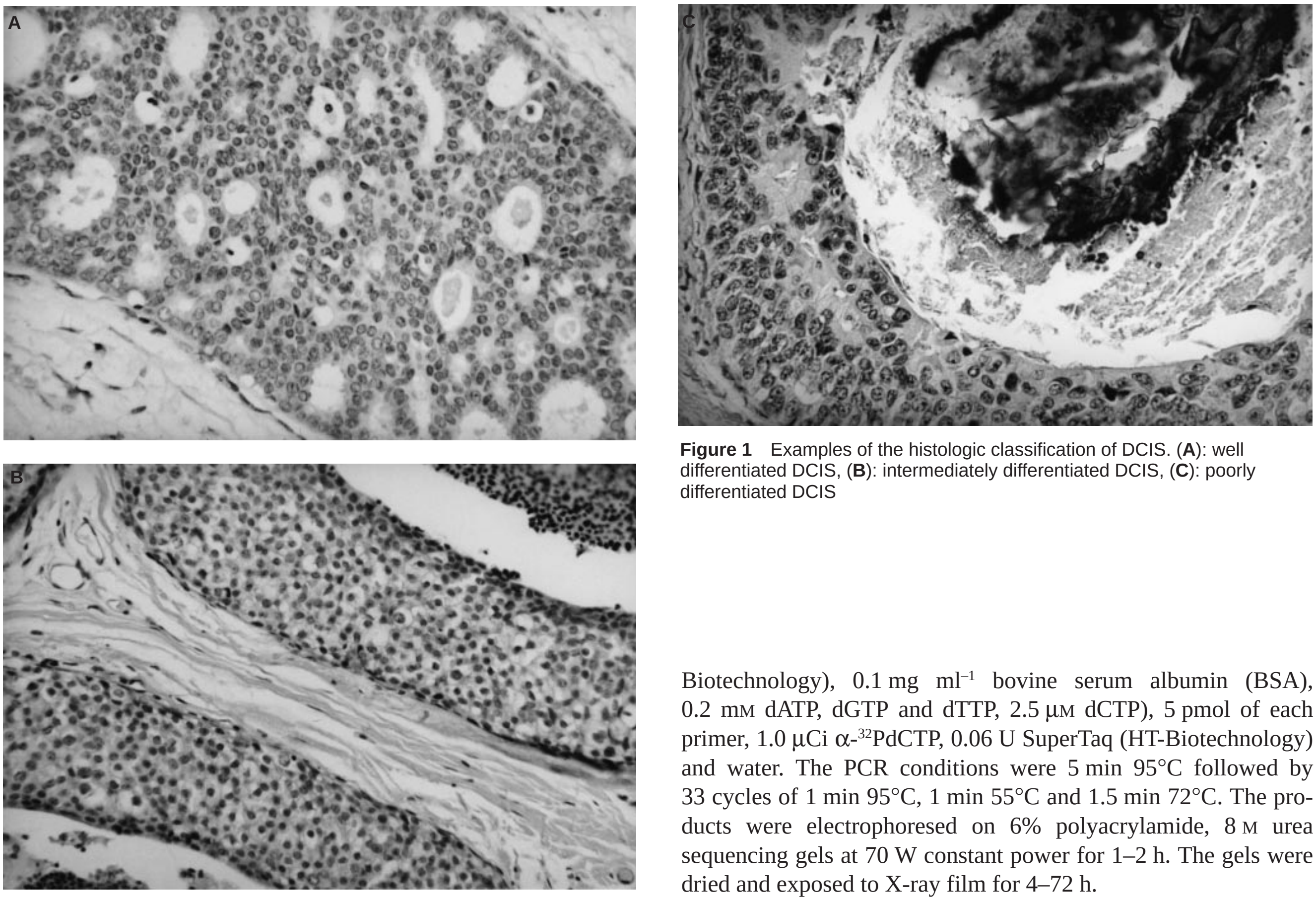

**Figure 1** Examples of the histologic classification of DCIS. (**A**): well differentiated DCIS, (**B**): intermediately differentiated DCIS, (**C**): poorly differentiated DCIS

56°C for 72 h. Each day, 15 µl proteinase K (10 mg ml$^{-1}$) was added. Subsequently phenol–chloroform extraction was performed followed by an ethanol precipitation in the presence of 2 µl glycogen (20 mg ml$^{-1}$) and 7.5 M $NH_4Ac$ (Isola et al, 1994). The DNA was diluted in 100–200 µl 10 mM Tris–HCl (pH = 7.6)/ 0.1 mM EDTA.

### *Allelotyping and microsatellite markers*

DNA from tumour cells and from normal cells was analysed using the polymerase chain reaction (PCR) with primers for di-, tri- or tetranucleotide repeats of known chromosomal location. PCR was performed in a 96-well microtitreplate in a 12-µl reaction volume containing 1.5 µl DNA, 1.2 µl 10 × SuperTaq buffer (HT Biotechnology), 0.1 mg ml$^{-1}$ bovine serum albumin (BSA), 0.2 mM dATP, dGTP and dTTP, 2.5 µM dCTP), 5 pmol of each primer, 1.0 µCi α-$^{32}$PdCTP, 0.06 U SuperTaq (HT-Biotechnology) and water. The PCR conditions were 5 min 95°C followed by 33 cycles of 1 min 95°C, 1 min 55°C and 1.5 min 72°C. The products were electrophoresed on 6% polyacrylamide, 8 M urea sequencing gels at 70 W constant power for 1–2 h. The gels were dried and exposed to X-ray film for 4–72 h.

Microsatellite analysis was performed at 76 loci covering all chromosome arms. Many of the loci were chosen because they are located at or near regions found to be involved in breast cancer. Loci studied by microsatellite analysis are listed in Table 1. All markers used are described in the Genome Database (GDB). Allelic loss was defined as loss of one allele in DCIS DNA compared to the constitutional DNA as determined by visual inspection of the autoradiograph, which was clear in most cases due to the enrichment for tumour cells. However, when a residual signal was seen, due to contaminating normal tissue or tumour heterogeneity, quantitative analysis on a Phosphor Imager (Molecular Dynamics) was used to determine the allelic imbalance factor (the ratio of allele1/allele2 of the control DNA and allele1/allele2 of the tumour DNA). An imbalance factor over 1.8 was considered as LOH. To confirm the results, PCR reactions were repeated for all cases showing LOH.

 © 1999 Cancer Research Campaign

**Table 3** Number of loci involved in LOH compared to histological classification of DCIS (*P*-value was calculated using the $\chi^2$ test)

| | DCIS | | |
|---|---|---|---|
| | **Well-differentiated** | **Intermediately differentiated** | **Poorly differentiated** |
| No LOH | 7 | 6 | 10 |
| 1–3 chromosomal loci involved | 16 | 10 | 14 |
| > 3 chromosomal loci involved | | | 7 |
| | | | *P*-value:0.004 |

**Table 4** Summary of chromosomal regions involved in 15 DCIS cases detected by CGH

| Tumour number | Copy gain regions | Deleted regions |
|---|---|---|
| Poorly differentiated | | |
| BT1253 | 17q22–24, 8q23–24 | 17p, 8p21–23 |
| BT1251 | 17q22–24 | |
| BT1265 | 1q, 8q, 16p, 17q12, 17q22–24 | 11q, 16q, 17p, 22q |
| BT1204 | chrom. 5, 8q, 12q13–22, 20q | 11q23–25, 17p, 22q13 |
| BT1255 | 17q12, 17q22–24, 6p12 | |
| BT1268 | chrom. X | 17p, 5q23, 6q21–22 |
| BT1213 | 11q13, 12q14–15, 5q | 17p |
| BT1222 | 1q, 6p23–24 | 17p, 9q34, 16q |
| BT1211 | 4q, Xq21, 2q | 17p, 16q |
| BT1224 | 8q, 11q13, 12q14–15, 17q12, 20q | 8p |
| Well-differentiated | | |
| BT1290 | 1q, 16p | 16q22–24, 22q |
| BT1445 | 1q, 3p24, 11q13, 19q | 3p12–14, 11q23–25 |
| BT1230 | chrom5, 7, 12, 19, 20 and X | |
| BT1285 | | 8p12–22, 16q, 19 |
| BT1238 | No changes | No changes |

### Gene amplification

### *Southern blot analysis*

From tissuebanks of the NCI and LUMC 80 samples of freshly frozen samples were collected. H&E sections of the frozen samples were examined by light microscopy and the tumour percentage was estimated. Only 32 specimens, which is a subset of the samples used for LOH analysis, met the requirement of 30% or more tumour cells and were classified as 20 poorly, six intermediately and six well-differentiated DCIS.

High molecular weight DNA was isolated by standard methods (Sambrook et al, 1989). In addition, the following cell lines and tissues were used as controls: two breast carcinoma cell lines, MDA134 and SKBR3 (c-*myc* and c-*erb*B2/*neu* gene amplification) and a cell line derived from normal mammary tissue (HBL100) and placenta. DNA was digested with the restriction endonuclease *Eco*RI (Pharmacia, Uppsala, Sweden) using buffers recommended by the suppliers. The digested DNA was size-fractionated overnight by gel electrophoresis on a 0.8% agarose gel in 1 × TAE, denatured and transferred with 1 M sodium chloride–0.4 M sodium hydroxide onto nylon membranes (Hybond $N^+$, Amersham, UK). After transfer the filters were neutralized in 0.5 M Tris–HCl–1 M sodium chloride (pH = 7.2) buffer, dried and baked at 80°C for 2 h.

### *Hybridization*

Filters were pre-hybridized for 0.5 h at 65°C in hybridization mix (1 M $Na_2HPO_4$–1 M $NaH_2PO$ (pH = 7.2)/7% SDS–0.5 M EDTA). The pSV2*erb*B2/*neu* construct which contains the complete cDNA of c-*erb*B2/*neu* was kindly provided by Dr Yamamoto (Yamamoto et al, 1986). The EGF receptor cDNA clone was kindly provided by Dr Ullrich (Ullrich et al, 1984). The c-*myc* probe was derived from the pHM-1 clone containing the complete human c-*myc* gene (Adams et al, 1985). From this clone a 400 bp *Pst*I/*Pst*I fragment of exon II was used for hybridization. The MDM2 probe was a 585 bp reverse transcription PCR (RT-PCR) fragment spanning nucleotides 650–1214 of the published cDNA sequence (Oliner et al, 1992; Marchetti et al, 1995). The *bek* and *flg* probes were kindly provided by Dr Theillet (Adnane et al, 1991). All probes were radiolabelled with 20 μCi of [$\alpha$-$^{32}$P]dCTP (> 3000 Ci $mmol^{-1}$, Amersham, UK) using a random-primed labelling kit (Pharmacia, Upsalla, Sweden). After at least 17 h of hybridization, filters were washed at 65°C to a final stringency of 0.1 × standard saline citrate (SSC) 0.1% SDS. The filters were exposed to Kodak Xomat AR films with DuPont Cronex Lightning Plus screens for 2–5 days at –70°C. The degree of amplification was determined by visual inspection comparing signal intensity of the various probes with probes located in non-amplified regions at the same chromosome arm (chromosome 8 (thyroglobulin), 12 (D12S2) and 17 (D17Z1)). All probes are described in the GDB. The same filters were repeatedly hybridized.

### CGH analysis

Five well-differentiated DCIS and ten poorly differentiated DCIS were tested by CGH. Tumour DNA (test DNA) was extracted

© 1999 Cancer Research Campaign

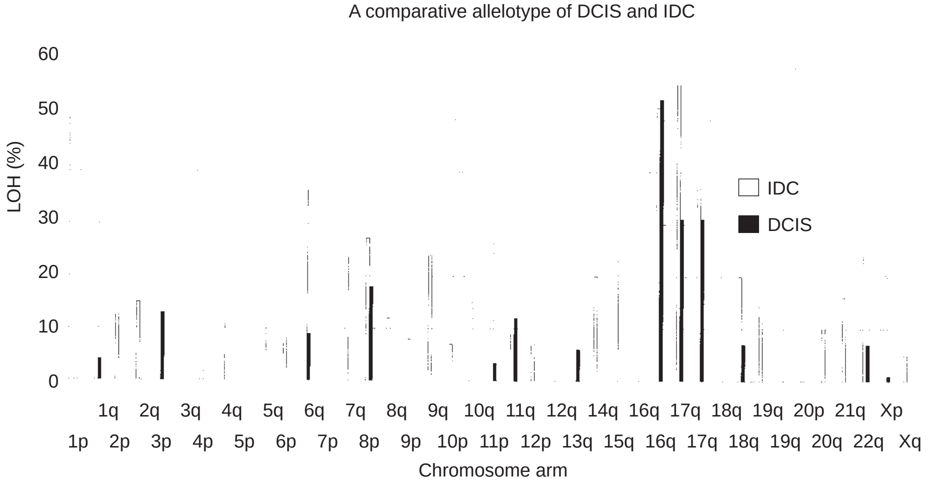

**Figure 2** Allelotype of DCIS (*n*=78) compared with invasive breast cancer. The allelotype of DCIS was constructed using our data; the allelotype of invasive breast cancer was constructed using data from the literature (Devilee et al, 1994)

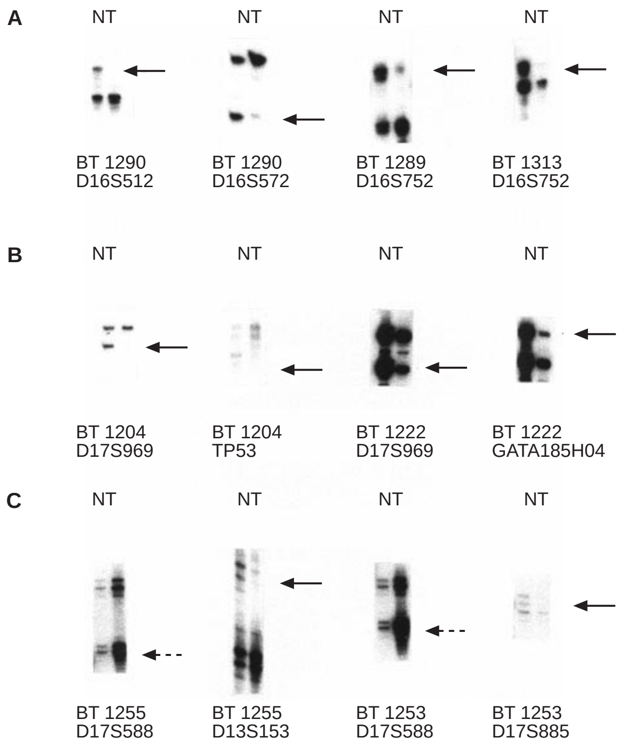

**Figure 3** Examples of allelic imbalance in DCIS. PCR products are shown from N (normal) and T (tumour) DNA from individuals with DCIS. The loss of one allele in the tumour lane is seen in all photographs showing ——>. Allelic gain is shown as ---->. (**A**) Representative microsatellite analysis of well differentiated DCIS showing LOH with markers on chromosome 16q. (**B**) Representative microsatellite analysis of poorly differentiated DCIS showing LOH with markers on chromosome 17p. (**C**) Two of the four poorly differentiated DCIS (BT1255 and BT1253) in which allelic gain occurs with marker D17S588 (17q23); for the same DCIS cases LOH is shown for D13S153 (13q12) and D17S855 (17q21)

either from frozen material (BT1253 and BT1290) or from microdissected, paraffin-embedded material (all other cases). DNA from uncultured peripheral blood from a healthy female was used as reference DNA. The CGH procedure was based on the protocol described by Kallioniemi (Kallioniemi et al, 1992, 1994), with a few modifications. Briefly, test DNA was direct-labelled with lissamine-dUTP (NEN Life Sciences) and reference DNA was labelled with fluorescein isothiocyanate (FITC)-dATP (duPont), both by nick-translation. Two hundred nanograms of each labelled DNA and 10 μg of COT1 DNA dissolved in 10 μl hybridization buffer (50% formamide 2 × SSC 10% dextran sulphate) were hybridized to normal male meta-phases and incubated at 37°C for 4 days. Post-hybridization washes were performed with 2 × SSC at 37°C (3 washes) followed by 0.1 × SSC at 60°C (3 washes). Slides were counterstained with 4,6-diamidino-2-phenylindole (DAPI) in an antifade solution (Vectashield, Vector).

Images were acquired with an epifluorescent microscope (Leica, DM) equipped with three single excitation filters, a multi-bandpass dichroic mirror, a multi-band pass emission filter (P-1 filter set, Chroma Technology, Brattleborough, VT, USA) and a cooled CCD camera (Photometrics Inc.). The green, red and blue images were collected sequentially by changing the excitation filter. Grey level images (12 bit) were saved using a routine build up in SCIL-Image (TNO, Delft, The Netherlands) and implemented on a Power Macintosh 8100.

For CGH analysis the QUIPS XL (Downers Grove, IL, USA) software from Vysis was used. For the profiles, losses of DNA sequences are defined as chromosomal regions where the mean green to red fluorescent ratio and its 95% confidence interval is below 0.9 while gains are defined as chromosomal regions where this ratio is above 1.1. These thresholds were based on measurements from a series of five controls. The heterochromatic, pericentromeric, and the telomeric regions of the chromosomes, as well as 1p32-pter were excluded from the analysis, because these regions show variable results in normal controls (Kallioniemi et al, 1994).

### RESULTS

### Allelotyping

The frequency of LOH for each marker is summarized in Table 1. Chromosome arms with the highest percentage of LOH are chromosome 3 (13%), 8 (18%), 11 (12%), 13q (12%), 16q (54%), 17p (21%), 17q (21%), lower percentages were found on chromosome

© 1999 Cancer Research Campaign

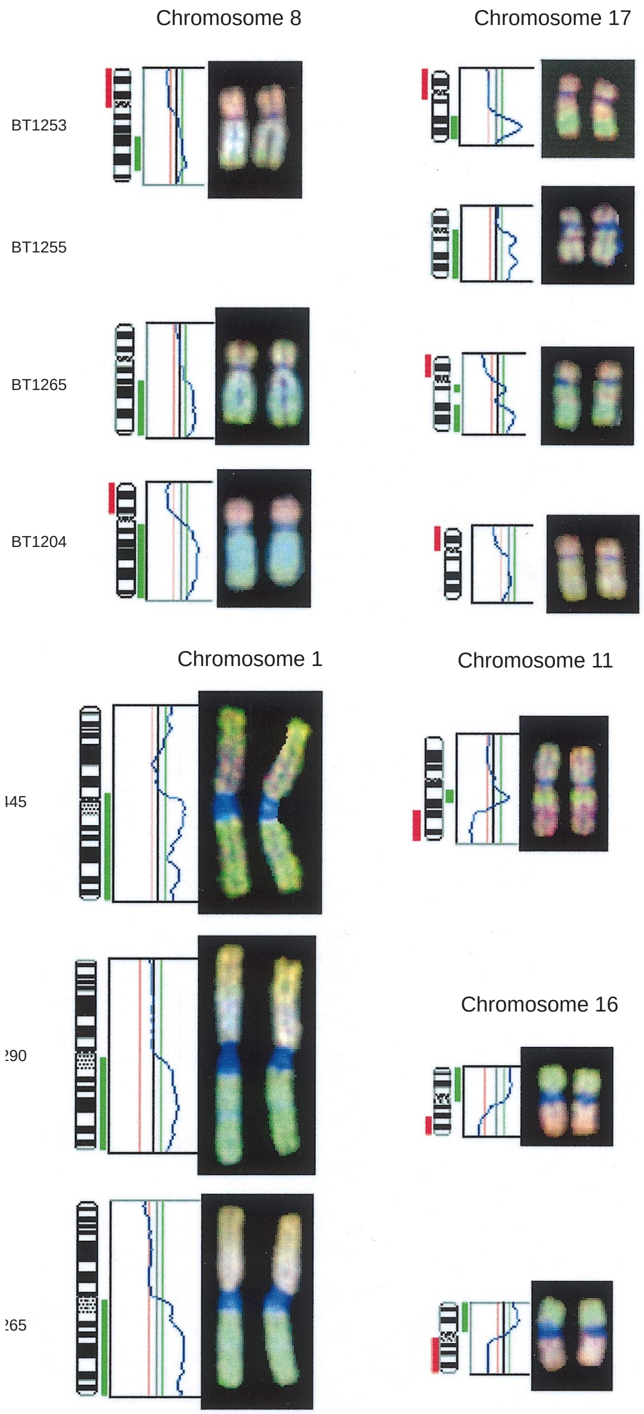

**Figure 4** (**A**) An illustration of the most common alterations found by CGH in poorly differentiated DCIS, showing amplification of the q-arm of chromosome 8, combined in two cases with deletion of the p-arm. Amplification of two regions on chromosome 17q, 17q12 and 17q22–24 is a predominant feature in poorly differentiated DCIS; in three cases there also was a deletion on chromosome 17p. (**B**) An illustration of the most common alterations detected by CGH in well differentiated DCIS, showing amplification of 1q, combined with a deletion of chromosome 16q and an amplification of 16p. In one case (BT1290) amplification of the 11q13 region was found

1q (4%), 6q (9%), 18q (7%), 22q (7%) and Xq (1%). No LOH was found on chromosome 2, 4, 5, 7, 9, 10, 12, 14, 15, 19, 20 and 21. A compiled allelotype was made from published studies on cases of invasive breast carcinoma (Devilee et al, 1994) in order to compare this with the DCIS allelotype (Figure 2). For all loci showing LOH in DCIS, LOH was seen in invasive breast cancer. However, LOH on chromosome arm 1p, 7q, 9q, 15q and 18p was found in > 20% of invasive carcinomas but not in DCIS suggesting a possible involvement of tumour suppressor genes in these regions in progression from DCIS to invasive breast cancer.

LOH on chromosome 16 and 17 were the most frequent findings in DCIS. Representative examples of LOH on chromosome 16 and 17 are shown in Figure 3A and 3B. Table 2 shows the association between histologic type of DCIS and the frequency of LOH on chromosome 16 and 17. LOH on chromosome 17 was found in 70% of the poorly differentiated DCIS and in 17% of the well-differentiated DCIS. In 21% (15/72) of DCIS (predominantly poorly differentiated) LOH was found restricted to the 17p-arm. In two poorly differentiated cases, LOH on 17p was restricted to the region 17p13.3. In all other cases the LOH region included the 17p13.1 region which contains the p53 gene.

Also in 21% (15/72) LOH was found on the 17q-arm. The common region of overlap was located between marker D17S579 and D17S855. In 10% of the cases (6/72) whole chromosome 17 was afflicted by LOH. On chromosome 16q an inverted picture was seen: in well- and intermediately differentiated DCIS, LOH was found in 66% (26/39) of the cases and in 39% (12/31) of the poorly differentiated DCIS. A total of 16 cases well-differentiated DCIS showed LOH on chromosome 16q. Of these, eight cases showed LOH for all markers informative on chromosome 16q, two cases showed LOH of the whole chromosome and six cases defined a smallest region of overlap between marker D16S2624 and D16S1320 (16q22.1–16q24.3). For both chromosome 16 and 17 the percentage of LOH in intermediately differentiated DCIS is 63%. In the four cases of well-differentiated DCIS with LOH on chromosome 17, also LOH on chromosome 16 was present. In the 12 cases of poorly differentiated DCIS that show LOH on 16q, 83% also showed LOH on chromosome 17. In poorly differentiated DCIS the number of loci involved in LOH per tumour is significantly higher than in intermediately or well-differentiated DCIS (Table 3).

Using marker D17S588 four cases of poorly differentiated DCIS (BT1251, 1253, 1255 and 1265) showed a pattern which we interpreted as allelic gain on chromosome 17q23. The same tumour samples showed on other loci a complete loss of one allele (Figure 3C) which indicates that these tumours exhibit a normal pattern of LOH for other loci.

### Oncogene amplification

Thirty-two cases were analysed using Southern blot hybridization with six oncogene probes. C-*erb*B2/*neu* gene amplification was found in 31% of the cases (10/32). C-*myc*, EGF-receptor, *bek*, *flg* and MDM2 were not amplified in any of these DCIS. All cases with oncogene amplification were of the poorly differentiated type.

### Comparative Genomic Hybridization

DNA from 15 cases of DCIS was subjected to CGH: ten were poorly differentiated and five well-differentiated (Table 4). All 15 cases had multiple genetic aberrations affecting three to seven different chromosomal regions per tumour. The most frequent changes in the ten cases of poorly differentiated DCIS were gains

© 1999 Cancer Research Campaign

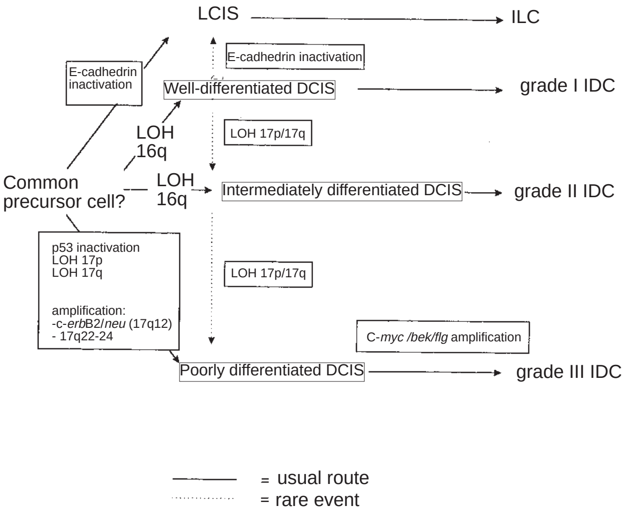

**Figure 5** Proposed model for breast cancer initiation and progression. The data to construe this model are from our own work and from the literature. We hypothesize that there are several pathways for the development of different types of breast cancer, which differ at the genetic level. A particular genetic alteration is not a prerequisite for developing a certain tumour type, but a strong correlation is found (e.g. LOH at 16q in well- and intermediately differentiated DCIS and LOH at 17 in poorly differentiated DCIS)

on 8q (4/10), 12q (3/10) and 17q (5/10) and losses on 16q (3/10) and 17p (6/10). For well-differentiated DCIS the most frequent alterations were gain on 1q (2/5) and loss on 16q (2/5) (Figure 4).

### Concordance between LOH, Southern blot analysis and CGH

In all cases we could confirm the deletions found by CGH with LOH analysis. However, not all loci with LOH showed deletions using CGH. An explanation can be that LOH frequently arises by deletion of one allele followed by duplication of the remaining allele as a result of mitotic recombination (Gupta et al, 1997). In four poorly differentiated DCIS (BT1251, 1253, 1255 and 1265) allelic imbalance for marker D17S588 was shown in the LOH analysis (Figure 3C). By CGH, amplification at the 17q22-24 locus, which is the region in which D17S588 is located, was found. In two out of four cases, co-amplification of 17q12, which contains the c-*erb*B2/*neu* locus, was found (BT1255 and BT1265). Copy gain of 17q12 found in three cases could always be confirmed by amplification of c-*erb*B2/*neu* in Southern blot analysis. In two cases (BT1251 and BT1253) only the 17q22-24 region was amplified (Table 4 and Figure 4) and also amplification of the 17q12 alone was found (BT1224). By Southern blot analysis no c-*myc* amplification was found in 32 cases of DCIS whereas using CGH 8q amplification was found in 40% (4/10) of the poorly differentiated DCIS (Table 4 and Figure 4). In all these cases an increased signal intensity was seen for the entire q-arm of the chromosome instead of a narrow amplification unit as is seen for amplifications on 17q12 and 17q22-24. This suggests that there is a duplication of the entire q-arm of one of the copies of chromosome 8 which is missed by Southern blotting because the reference probe is also located on 8q. By Southern blot analysis no amplification was found for MDM2 (located on 12q13). By CGH we found amplification restricted to the q14–q15 region of chromosome 12 in 30% of the poorly differentiated DCIS. The EGF-receptor (7q26), *bek* (10q26) and *flg* (8p12) was never found to be amplified, neither by Southern blot nor CGH.

### DISCUSSION

The aim of this comprehensive study was to gain insight in the locations and frequencies of regional chromosomal alterations in different histological types of DCIS of the breast. Malignant tumours arise through a cascade of genetic events involving oncogenes and tumour suppressor genes resulting in a disruption of normal cell growth regulation. Additional mutations and deletions result in progression to invasion and metastasis. We have studied whether there is a genetic explanation for the histologically different types of DCIS.

In our study we found that LOH on chromosome 16q was found in 66% of well- and intermediately differentiated DCIS and only in 39% of poorly differentiated DCIS. On 16q22.1, the E-cadherin gene is located which has previously found to be inactivated in invasive lobular carcinoma of the breast (Kanai et al, 1994; Berx et al, 1995). Recently, we have shown that in lobular carcinoma in situ (LCIS) E-cadherin is inactivated, whereas all cases of DCIS express E-cadherin at the cell membrane (Vos et al, 1997). Therefore another yet unidentified tumour suppressor gene is target of LOH on 16q in DCIS (Cleton-Jansen et al, 1994; Tsuda et al, 1994).

On chromosome 17, LOH was found in 70% of the poorly differentiated DCIS cases, versus 17% in well-differentiated DCIS. In 21% (15/72) (predominantly poorly differentiated DCIS) LOH is found on the 17p-arm only. In most cases, p53 is thought to be the target gene on 17p. Mutations in p53 are present in DCIS as has been found by us (data not shown) and others (Chitemerere et al, 1996; Munn et al, 1996).

We and others (Munn et al, 1996) found LOH in the 17q21 region in 21% and 67% of the DCIS cases respectively. LOH on 17q occurs in a large proportion of invasive breast cancers, both in familial and sporadic cases. Chromosomal deletions on 17q21 point to a tumour suppressor gene close to the BRCA1 gene. Inactivating point mutations in BRCA1 have only been found in the germline in familial breast cancer, not as a de novo mutation in sporadic breast cancer (Miki et al, 1994). Three other studies have previously identified chromosomal loci with frequent allelic loss in DCIS. In a study by Radford (Radford et al, 1995) the highest percentage of LOH was found on chromosome 8p, 13q, 16q, 17p and 17q. Aldaz (Aldaz et al, 1995) reported frequent LOH on 16q, 17p and 17q. In these studies DCIS was not histologically classified, but they confirm that alterations on chromosome 16 and 17 are early events in breast carcinogenesis. The only study (Fujii et al, 1996) in which histological type and LOH were correlated shows that loss on chromosome 16q and 17p were common in all histologic types of DCIS, whereas loss on other chromosome arms was uncommon in low-grade compared to intermediate- and high-grade DCIS. As in our study the number of loci with LOH is higher in poorly differentiated DCIS than in intermediately or well-differentiated DCIS. However, in contrast to other findings we were able to make a genetic distinction between well- and poorly differentiated DCIS based on LOH and CGH profiles. It is presently unclear whether breast cancer development via an intermediately differentiated DCIS represents a distinct genetic pathway. It is likely that some cases of intermediately differentiated DCIS are derived from well-differentiated DCIS that show an increase in cytonuclear pleomorphism as a result of the

 *© 1999 Cancer Research Campaign*

accumulation of additional genetic alterations. During the development of a histological classification for DCIS (Holland et al, 1994) it has not been possible to classify all DCIS without having a category of intermediately differentiated DCIS. When more specific tumour suppressor genes are cloned in the future, it will become clear whether there are specific genetic alterations in intermediately differentiated DCIS.

Amplification of oncogenes is another important genetic alteration in breast cancer. The most notable gain we found was 17q amplification, for which at least two separate regions can be discriminated, 17q12 and 17q22–q24. The oncogene that is amplified in the 17q12 region is *c-erb*B2/*neu*. The 17q22–q24 locus harbours a yet unknown oncogene(s) which has been found to be amplified in breast cancer cell lines and invasive breast cancer by CGH (Kallioniemi et al, 1994).

In 10–15% of the invasive breast cancers amplification of the c-*myc* gene on chromosome 8q24 is found (Varley et al, 1987; Berns et al, 1992). We could not detect c-*myc* amplification in DCIS by Southern blot analysis. Using CGH, gain of the entire q-arm of chromosome 8 was found. We hypothesize that duplication of the q-arm of chromosome 8 explains these results and conclude that amplification of the c-*myc* gene occurs in a later stage of breast cancer progression.

In conclusion, our findings support the evidence of different genetic routes to develop a specific type of carcinoma in situ of the breast. Figure 5 shows our proposal for a genetic model for the development of DCIS and progression to invasive breast cancer based on the data reported by us and others. Inactivation of E-cadherin is the predominant event in LCIS and ILC. LOH on 16q is found in the majority of well-differentiated DCIS and since E-cadherin expression is always detectable in DCIS, there must be a yet unknown tumour suppressor gene responsible for the development of this type of DCIS. LOH and amplification of several regions on chromosome 17 are predominant in poorly differentiated DCIS, indicating that (multiple) aberrations on chromosome 17 are responsible for this subtype of DCIS. Inactivation of p53 leads to genomic instability including gene amplification (Livingstone et al, 1992) and may explain the appearance of gene amplification in combination with a high incidence of LOH on 17 in poorly differentiated DCIS. In most cases progression from well-differentiated DCIS to Grade I invasive carcinoma and poorly differentiated DCIS to Grade III invasive will take place. In rare cases progression from well- to intermediately or intermediately to poorly differentiated DCIS is possible and associated with genetic alterations on chromosome 17. In general, various alterations on chromosome 16 and 17 are early and crucial steps in the development of the different histological types of DCIS.

### ACKNOWLEDGEMENTS

We thank Dr R Holland, University Hospital Nijmegen, for contributing a number of the DCIS cases used in this study. This work was supported by the Dutch Cancer Society (KWF 94-757).

### REFERENCES

Adams JM, Harris AW, Pinkert CA, Corcoran LM, Alexander WS, Cory S, Palmiter RD and Brinster RL (1985) The c-*myc* oncogene driven by immunoglobulin enhancers induces lymphoid malignancy in transgenic mice. *Nature* **318**: 533–538

Adnane J, Gaudray P, Dionne CA, Crumley G, Jaye M and Schlessinger J (1991) BEK and FLG, two receptors to the members of the FGF family are amplified in subsets of human breast cancers. *Oncogene* **6**: 659–663

Aldaz CM, Chen T, Sahin A, Cunningham J and Bondy M (1995) Comparative allelotype of *in situ* and invasive human breast cancer high frequency of microsatellite instability in lobular breast carcinomas. *Cancer Res* **55**: 3976–3981

Berns EMJJ, Klijn JGM, van Putten WLJ, van Staveren IL, Portengen H and Foekens JA (1992) C-*myc* amplification is a better prognostic factor than HER-2/neu amplification in primary breast cancer. *Cancer Res* **52**: 1107–1113

Berx G, Cleton-Jansen A-M, Nollet F, de Leeuw WJF, van de Vijver MJ, Cornelisse CJ and van Roy F (1995) E-cadherin is a tumour/invasion suppressor gene mutated in human lobular breast cancers. *EMBO* **14**: 6107–6115

Berx G, Cleton-Jansen A-M, Strumane K, de Leeuw WJF, Nollet F, Van Roy F and Cornelisse CJ (1996) E-cadherin is inactivated in a majority of invasive human lobular breast cancers by truncation mutations throughout its extracellular domain. *Oncogene* **13**: 1919–1925

Bobrow LG, Happerfield LC, Gregory WM, Springall RD and Millis RR (1994) The classification of ductal carcinoma in situ and its association with biological markers. *Semin Diagn Pathol* **11**: 199–207

Chitemerere M, Andersen TI, Holm R, Karlsen F, Borresen AL and Nesland JM (1996) TP53 alterations in atypical ductal hyperplasia and ductal carcinoma in situ of the breast. *Breast Cancer Res Treat* **41**: 103–109

Cleton-Jansen A-M, Moerland EW, Kuipers-Dijkshoorn NJ, Callen DF, Sutherland GR, Hansen B, Devilee P and Cornelisse CJ (1994) At least two different regions are involved in allelic imbalance on chromosome 16q in breast cancer. *Genes Chromosomes Cancer* **9**: 101–107

Coene ED, Schelfhout V, Winkler RA, Schelfhout AM, van Roy N, Grooteclaes M, Speleman F and de Potter CR (1997) Amplification units and translocation at chromosome 17q and *c-erb*B2 overexpression in the pathogenesis of breast cancer. *Virchows Arch* **430**: 365–372

Devilee P and Cornelisse CJ (1994) Somatic changes in human breast cancer. *Biochimica Biophysica Acta* **1198**: 113–130

Done SJ, Arneson NCR, Özçelik H, Redston M and Andrulis IL (1998) p53 mutations in mammary ductal carcinoma in situ but not in epithelial hyperplasias. *Cancer Res* **58**: 785–789

Fujii H, Szumel R, Marsh C, Zhou W and Gabrielson E (1996) Genetic progression, histological grade, and allelic loss in ductal carcinoma in situ of the breast. *Cancer Res* **56**: 5260–5265

Gupta PK, Sahota A, Boyadjiev SA, Bye S, Shao C, O'Neill JP, Hunter TC, Albertini RJ, Stambrook PJ and Tischfield JA (1997) High frequency in vivo loss of heterozygosity is primarily a consequence of mitotic recombination. *Cancer Res* **57**: 1188–1193

Holland R, Peterse JL, Millis RR, Eusebi V, Faverly D, van de Vijver MJ and Zafrani B (1994) Ductal carcinoma in situ: a proposal for a new classification. *Semin Diagn Pathol* **11**: 167–180

Isola J, de Vries S, Chu L, Ghazvini S and Waldman F (1994) Analysis of changes in DNA sequence copy number by comparative genomic hybridization in archival paraffin embedded tumour samples. *Am J Pathol* **145**: 1301–1308

James LA, Mitchell ELD, Menasce L and Varley JM (1997) Comparative genomic hybridization of ductal carcinoma in situ of the breast: identification of regions of DNA amplification and deletion in common with invasive breast carcinoma. *Oncogene* **14**: 1059–1065

Kallioniemi A, Kallioniemi OP, Sudar D, et al (1992) Comparative genomic hybridization for molecular cytogenetic analysis of solid tumours. *Science* **258**: 818–821

Kallioniemi OP, Kallioniemi A and Piper J (1994) Optimizing comparative genomic hybridization for analysis of DNA sequence copy number changes in solid tumours. *Genes Chromosomes Cancer* **10**: 231–243

Kanai Y, Oda T, Tsuda H, Ochiai A and Hirohashi S (1994) Point mutation of the E-cadherin gene in invasive lobular carcinoma of the breast. *Japanese Journal of Cancer Research* **85**: 1035–1039

Kuukasjarvi T, Tanner M, Pennanen S, Karhu R, Kallioniemi O-P and Isola J (1997) Genetic changes in intraductal breast cancer detected by comparative genomic hybridization. *Am J Pathol* **150**: 1465–1471

Livingstone LR, White A, Sprouse J, Livanos E, Jacks T and Tlsty TD (1992) Altered cell cycle arrest and gene amplification potential accompany loss of wild-type p53. *Cell* **70**: 923–935

Mack L, Kerkvliet N, Doig G and O'Malley FP (1997) Relationship of a new histological categorization of ductal carcinoma in situ of the breast with size and the immunohistochemical expression of p53, c-erbB2, BCL2 and Ki-67. *Hum Pathol* **28**: 974–979.

Marchetti A, Buttitta F, Girlando S, Dalla Parma P, Pellegrini S, Fina P, Doglioni C, Belilacqua G and Barbareshi M (1995) MDM2 alterations and mdm2 protein overexpression in breast carcinomas. *J Pathol* **175**: 31–38.

© 1999 Cancer Research Campaign

Miki Y, Swensen J, Shattuck-Eidens D, Futreal PA, Harshman K et al. (1994) A strong candidate for the breast and ovarian cancer susceptibility gene BRCA1. *Science* **266**: 66–71
Munn KE, Walker RA, Menasce L and Varley JM (1996) Mutations of the TP53 gene and allelic imbalance at chromosome 17p13 in ductal carcinoma in situ. *Br J Cancer* **74**: 1578–1585
Munn KE, Walker RA, Menasce L and Varley JM (1996) Allelic imbalance in the region of the BRCA1 gene in ductal carcinoma *in situ* of the breast. *Br J Cancer* **73**: 636–639
Murphy DS, Hoare SF, Going JJ, Malon EE, George WD, Kaye SB, Brown R, Black DM and Keith WN (1995) Characterization of extensive genetic alterations in ductal carcinoma in situ by fluorescence in situ hybridization and molecular analysis. *J Nat Cancer Inst* **87**: 1694–1704
Oliner JD, Kinzler KW, Melzer PS, George D and Vogelstein B (1992) Amplification of a gene encoding a p53 associated protein in human sarcomas. *Nature* **358**: 80–83
Radford DM, Fair KL, Phillips NJ, Ritter JH, Steinbrueck T, Holt MS and Donis-Keller H (1995) Allelotyping of ductal carcinoma *in situ* of the breast: Deletion of loci 8p, 13q, 16q, 17p and 17q. *Cancer Res* **55**: 3399–3405
Sambrook J, Fritsch EF and Maniatis T (1989) *Molecular Cloning: a Laboratory Manual*. Cold Spring Harbor Laboratory Press: New York
Scott MA, Lagios MD, Axelsson K, Rogers LW, Anderson TJ and Page DL (1997) Ductal carcinoma *in situ* of the breast: reproducibility of histological subtype analysis. *Hum Pathol* **28**: 967–973
Silverstein MJ, Poller DN, Waisman JR, Colburn WJ, Barth A, Gierson ED, Lewinsky B, Gamagami P and Slamon DJ (199?) Prognostic classification of breast ductal carcinoma in situ. *Lancet* **345**: 1154–1157
Simpson JF, Quan DE, O'Malley F, Odom-Maryon T and Clarke PE (1997) Amplification of CCND1 and expression of its protein product, cyclin D1, in ductal carcinoma *in situ* of the breast. *Am J Pathol* **151**: 161–168
Tavassolli FA (1992) *Pathology of the Breast*. Appleton & Lange: Norwalk CT
Tsuda H, Callen DF, Fukutomi T, Nakamura Y and Hirohashi S (1994) Allele loss on chromosome 16q24.2-ter occurs frequently in breast cancers irrespective of differences in phenotype and extent of the spread. *Cancer Res* **54**: 513–517
Ullrich A, Coussens L, Hayflick JS, Dull TJ, Gray A, Tam AW, Lee J and Yarden Y (1984) Human epidermal growth factor receptor cDNA sequence and aberrant expression of the amplified gene in A431 epidermoid carcinoma cells. *Nature* **309**: 418–425
Varley JM, Swallow JE, Brammar WJ, Whittaker JL and Walker RA (1987) Alterations to either c-erbB2(neu) or c-myc proto-oncogenes in breast carcinomas correlate with poor short-term prognosis. *Oncogene* **1**: 423–430
Vogelstein B, Fearon ER, Hamilton SR, Kern SE, Preisinger AC, Leppert M, Nakamura Y, White R, Smits AM and Bos JL (1988) Genetic alterations during colorectal tumour development. *N Engl J Med* **319**: 525–532
Vos CBJ, Cleton-Jansen A-M, Berx G, de Leeuw WJF, ter Haar NT, Peterse JL, Cornelisse CJ and van de Vijver MJ (1997) E-cadherin inactivation in lobular carcinoma *in situ* of the breast: an early event in tumourigenesis. *Br J Cancer* **76**: 1131–1133
Yamamoto T, Ikawa S, Akiyama T, Semba K, Nomura N, Miyajima N, Saito T and Toyoshima K (1986) Similarity of protein encoded by the human c-*erb*-B2 gene to epidermal growth factor receptor. *Nature* **319**: 230–234
Zafrani B, Leroyer A, Fourquet A, Laurent M, Trophilme D, Validire P and Sastre-Garau X (1994) Mammographically detected ductal *in situ* carcinoma of the breast analyzed with a new classification. A study of 127 cases: correlation with estrogen and progesterone receptors, p53 and c-*erb*B2 proteins and proliferative activity. *Semin Diagn Pathol* **11**: 208–214

*© 1999 Cancer Research Campaign*
